# Circular RNA circFOXP1 promotes angiogenesis by regulating microRNA -127-5p/CDKN2AIP signaling pathway in osteosarcoma

**DOI:** 10.1080/21655979.2021.1989258

**Published:** 2021-11-27

**Authors:** Haiping Zhang, Ziliang Yu, Bingbing Wu, Farui Sun

**Affiliations:** a Department of Orthopedics, The Second Affiliated Hospital of Nantong University, Nantong, China; b Department of Orthopedics, Huangshi Central Hospital of East Hubei Medical Group Affiliated to Hubei Institute of Technology, Huangshi, China

**Keywords:** circFOXP1, miR-127-5p, CDKN2AIP, angiogenesis, osteosarcoma

## Abstract

Osteosarcoma is known to have a high metastatic potential, which is closely related to angiogenesis. circRNAs are closely associated with osteosarcoma metastasis. This study aims to investigate the role of Circular RNA circFOXP1 in angiogenesis in osteosarcoma. We detected circFOXP1 expression in osteosarcoma, as well as its prognostic value. Tube formation assay and immunohistochemistry staining were conducted to determine the condition of tube formation. RT-qPCR was performed to explore targeted genes. Luciferase reporter assays were carried out to explore the interaction between miR-127-5p, ircFOXP1, and CDKN2AIP, respectively. *In vivo* studies further confirmed the relationship between circFOXP1 and tumor angiogenesis in osteosarcoma. We found that circFOXP1 expression was increased in osteosarcoma, and could promote angiogenesis in osteosarcoma through upregulating CDKN2AIP expression. Moreover, circFOXP1 could directly bind to miR-127-5p, which further targets CDKN2AIP directly. In conclusion, circFOXP1 promoted angiogenesis by regulating miR-127-5p/CDKN2AIP signaling pathway in osteosarcoma.

## Introduction

Osteosarcoma is a common primary malignancy in children and adolescents, commonly with early symptoms of pain and high incidence. Currently, the standard therapy includes surgery and chemotherapy [1]. Despite great efforts, the efficacy is quite limited. The patients remain to have poor prognosis, with 5-year survival rate of less than 30% [2]. High metastatic potential is always considered to be the principal characteristic of osteosarcoma, which mainly leads to poor prognosis. Therein, the angiogenesis is quite essential for metastasis in osteosarcoma [3] and is a multistep process including cellular migration, vascular tube formation, and et al. [4], which resulting in abnormally formed and organized vessels with increased permeability and promoting tumor growth and metastases [5]. Therefore, anti-angiogenesis therapy might be a promising approach for osteosarcoma, rendering exploring the underlying mechanism of angiogenesis quite important.

circRNAs are characterized by a circular structure linked to covalent bonds, which renders them more stable than linear RNA. circRNAs are also highly conservative and are lack of 5′-cap and 3′-polyA tails, which is different from other non-coding RNAs [6]. In recent years, more and more studies have shown that circRNAs can play a key role in regulating angiogenesis in cancer. circRNA 100146 was found to promote angiogenesis through direct binding to miR-615–5p in NSCLC [7]. Hsa_circ_0007534 was detected to be significantly upregulated in breast cancer, and could promote angiogenesis by the combination with miR-593 as a sponge to upregulating MUC19 expression [8]. CircNF1 was also reported to be involved in angiogenesis by acting as the sponge of miR-16 in gastric cancer [9]. Although the research of circRNAs has progressed recently, circRNAs-function research requires further investigation compared to other RNAs for that understanding the functions and mechanisms of circRNAs might be useful in the prevention and treatment of cancer in the future.

Collaborator of alternative reading frame (CARF) is commonly referred to as CDKN2A Interacting Protein (CDKN2AIP) and is initially identified as the binding partner of ARF. CDKN2AIP is located in chromosome 4 (4q35) and has a ubiquitous expression, consisting of 580 amino acids [10]. Actually, CDKN2AIP has been observed to be upregulated in multiple cancers, especially in metastatic cancer [11]. Some studies also found that amplified CDKN2AIP could enhance angiogenesis. Higher CDKN2AIP expression was closely associated with higher expression of several markers involved in angiogenesis and metastasis [12]. Furthermore, CDKN2AIP has been reported to be targeted by miRNAs, and play extensive regulatory roles in cancer, such as miR-451 [11] and miR-335 [13]. However, the mechanism of CDKN2AIP in the regulation of angiogenesis in cancer remains unclear. Here, we hypothesize that circRNAs play a role in osteosarcoma angiogenesis and aim to investigate the relationship between circRNAs and CDKN2AIP, and their effects on angiogenesis in osteosarcoma. We found that circFOXP1 promotes angiogenesis by regulating miR-127-5p/CDKN2AIP signaling pathway in osteosarcoma. These observations offer circFOXP1 as a potential biomarker and target for treatment in osteosarcoma. The analysis of the molecular communication involved will therefore provide a novel understanding of the characteristics of tumor progression.

## Material and methods

### Clinical specimens and data

Forty cases of paired osteosarcoma and normal tissues (mean age: 40–65 years old) were chosen and confirmed by two pathologists. The consent was received from the study participants prior to the study commencement. This study was approved by the medical ethics committee of the Second Affiliated Hospital of Nantong University. Inclusion criteria: patients without radiotherapy, chemotherapy and immunotherapy. Exclusion criteria: metastatic osteosarcoma; preoperative chemotherapy and radiotherapy; with other types of malignant tumors.

### Cell lines

Osteosarcoma cell lines (U2OS, MG-63, HOS, and SAOS-02) and human osteoblast cell line (hFOB1.19) were purchased from ATCC. All the cells were cultured in DMEM medium (Gibco, USA) containing 10% fetal bovine serum with 1% penicillin and streptomycin (containing 100 u/ml penicillin and 100 μg/ml streptomycin) (Gibco, USA). All the cells were cultured at 37°C and 5% CO_2_.

### Quantitative real-time PCR

RNAs were extracted with Trizol (Takara, Japan). Then, Takara primescript RT Reagent kit with gDNA eraser kit was used for reverse transcription. SYBR Green method was used for quantitative real-time PCR (qRT-PCR). 2^−ΔΔCt^ method was used to determine fold changes.

### Western blot

Cells were collected at indicated time and were lysed with RIPA protein lysate buffer, and then were separated by SDS-polyacrylate gel electrophoresis. Protein strip was transferred to PVDF membrane, which was incubated with CDKN2AIP and GAPDH primary antibodies (1: 1000), secondary antibodies, and ECL luminescent solution, respectively.

### Cell transfection

The complex of Opti-MEM serum-free medium, diluted lipofectamine 2000, circFOXP1 siRNA, CDKN2AIP siRNA and miR-127-5p was incubated for 20 min at room temperature. Osteosarcoma cells were inoculated into 6-well plate. When cells reached 50% confluence, lipofectamine 2000-siRNA or mimic were added to the culture, which was replaced after 24 h. Cells were collected after 48 h for further research. circFOXP1 siRNA (5′-TTTTCCCTTTCCAAGGGCACAG-3′), CDKN2AIP siRNA (5ʹ-CGGAGUACCUGAGCCAGAAUT-3ʹ) or miR-127-5p mimic (5′-UAGUCUCGGG AGACUCGAAGUC-3ʹ) were purchased (GenePharma, China). siRNA transfection was performed following standard protocols [14].

### Tube formation assay

Tube formation assay was performed following standard protocols [15]. Dissolved Matrigel matrix (BD, USA) was incubated at 37°C for 30 min in the well. Osteosarcoma cells (2 × 10^4^/well) were seeded into 96‐well plates, which were coated with basement membrane in advance. The tube formation rate was determined by counting the tube‐like structures photographed by the microscope in each well.

### Immunohistochemistry staining

Tumor tissues were collected for immunohistochemistry staining. The primary antibody anti-CD31 (ab28364, 1:200, Abcam) was incubated overnight at 4°C and HRP‐ coupled secondary antibody (1:10,000, sc-2005, Santa Cruz) was incubated for 0.5 h at room temperature. Immunostaining images were photographed by the microscope.

### Animal experiments of tumor models

BALB/c nude mice (6 weeks) were purchased from Shanghai Sippr-BK Laboratory Animal Co. Ltd. Osteosarcoma cells were suspended in 100 μl free serum medium and injected subcutaneously into the right forelimb of mice. The length and the width of the tumor were measured every week. Tumor volume was calculated using the formula (Volume = 0.5 × length × width^2^). Mice were anesthetized with isoflurane gas and sacrificed by cervical dislocation 35 days after injection. All experiments were followed by Guidelines for Ethical Review of Laboratory Animal Welfare (GB/T 35892–2018) and were approved by the animal protection and ethics committee of the Second Affiliated Hospital of Nantong University.

### Luciferase reporter assay

In 293 T cells, the experiments tested the combination of miR-127-5p and circFOXP1 with circFOXP1 plasmid, circFOXP1 + miR-127-5p-mimic, circFOXP1 mutation plasmid, circFOXP1 mutation plasmid + miR-127-5p-mimic, following standard protocols [14]. The experiments tested CDKN2AIP is miR-127-5p targeted gene with CDKN2AIP plasmid, miR-127-5p-mimic + CDKN2AIP plasmid, CDKN2AIP mutant plasmid, miR-127-5p-mimic + CDKN2AIP mutant plasmid. After 24 h of transfection, the cells were lysed for centrifugation of 10,000 g for 5 min. The activities of Renilla and firefly luciferase were measured to calculate the ratio.

### Statistical analysis

Software SPSS 20.0 was applied for statistical analysis. The data were expressed with mean standard deviation, and the differences between groups were analyzed by t-test and one-way ANOVA. χ^2^ test was used for the comparison between groups of counting data. Kaplan-Meier was used for the survival curve. The difference was statistically significant (p < 0.05).

## Results

### circFOXP1 was negatively correlated with the survival rate in osteosarcoma

circRNA is crucial in promoting tumor angiogenesis, and we hypothesize that circ FOXP1 played role in osteosarcoma angiogenesis. In this work, we found that circFOXP1 is overexpressed in osteosarcoma and promotes the growth and angiogenesis of osteosarcoma via regulating miR-127-5p/CDKN2AIP axis.

**Figure 1.**
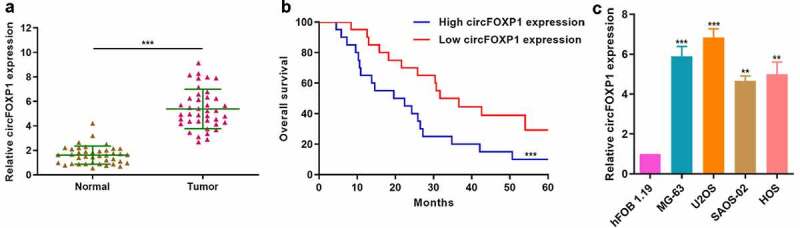
circFOXP1 is closely associated with poor prognosis in osteosarcoma. (a) circFOXP1 expression was measured in 40 osteosarcoma tissues and 40 noncancerous tissues via qRT-PCR. ***P < 0.001. (b) Kaplan-Meier survival analysis was performed to investigate the overall survival of patients in circFOXP1 high (N = 20)/low (N = 20) groups. ***P < 0.001. (c) circFOXP1 expression was determined in different osteosarcoma cell lines (U2OS, MG-63, HOS, and SAOS-02) and in human osteoblast cell line (hFOB1.19) using qRT-PCR. **P < 0.01, ***P < 0.001 vs hFOB1.19 group

To explore the role of circFOXP1 in osteosarcoma, we firstly measured circFOXP1 expression in osteosarcoma and normal tissues. circFOXP1 expression significantly increased in osteosarcoma compared to normal tissues (Figure 1a). Then, we divided the patients into high- and low-circFOXP1 expression group for survival analysis and found that 5-year survival rate of low-expression group was significantly higher than that of high-expression group (Figure 1b). Moreover, we explored circFOXP1 expression in osteosarcoma cell lines (U2OS, MG-63, HOS, and SAOS-02) and in human osteoblast cell line (hFOB1.19), and confirmed upregulated circFOXP1 expression in osteosarcoma cell lines (Figure 1c). We demonstrated that circFOXP1 was negatively correlated with the survival rate of osteosarcoma.

### circFOXP1 could promote the growth and angiogenesis in osteosarcoma

**Figure 2.**
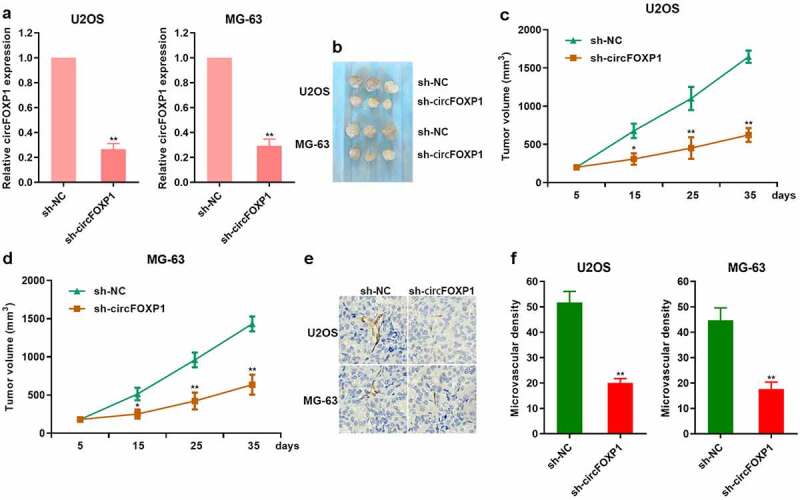
circFOXP1 promotes tumor growth and angiogenesis in osteosarcoma. (a) circFOXP1 expression was detected through qRT-PCR in MG-63 and U2OS cells treated with a lentivirus-based shRNA targeting FOXP1. **P < 0.01. (b–e) *In vivo* studies were performed with U2OS and MG-63 cells. Typical xenograft images (b). The tumor volume (c, d). *P < 0.05; **P < 0.01. (e–f) Immunohistochemistry was performed to measure CD31 expression in MG-63 and U2OS tumors (f). The microvascular density in tumor xenografts was assessed (g–h). **P < 0.01

To further study the effect of circFOXP1 on the angiogenesis in osteosarcoma, we obviously inhibited circFOXP1 expression in U2OS and MG-63 cells treated with a lentivirus-based shRNA targeting FOXP1 (Figure 2a). Then, U2OS and MG-63 cells treated with a lentivirus-based shRNA targeting FOXP1 were subcutaneously injected into nude mice. The tumor volume, which was comprised of cells treated with sh-circFOXP1, was smaller than the control one in both U2OS and MG-63 cell models (Figure 2b-d). It was observed that CD31 expression was decreased in tumor comprised of sh-circFOXP1 cells (Figure 2e). Similarly, the microvascular density was also lower in tumors comprising sh-circFOXP1 cells (figure 2f). These results suggested that circFOXP1 could promote the growth and angiogenesis of osteosarcoma.

### circFOXP1 promoted CDKN2AIP expression

**Figure 3.**
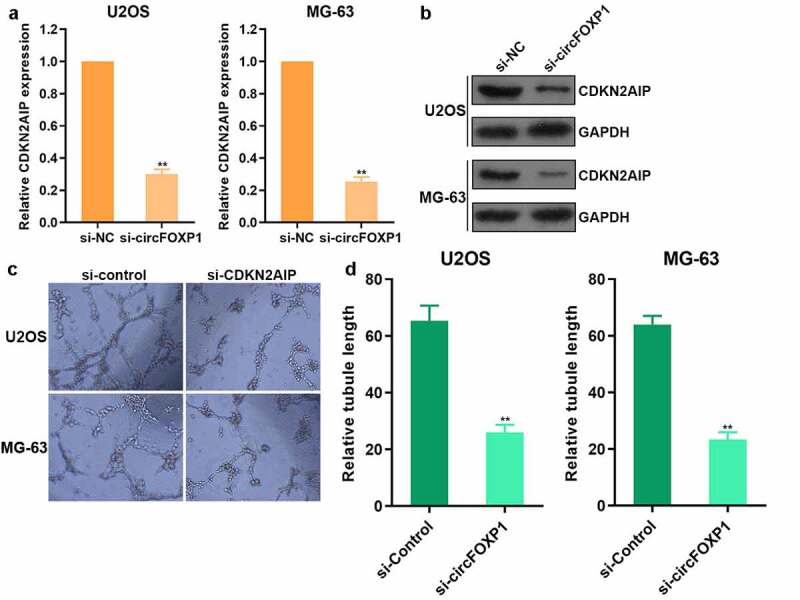
circFOXP1 promoted CDKN2AIP expression. (a-b) The expression of CDKN2AIP mRNA was detected by qRT-PCR (a) and CDKN2AIP protein was detected by western blot (b) when cells treated with circFOXP1 siRNA. (c-d) Tube formation assay was detected after CDKN2AIP siRNA (c) and circFOXP1 siRNA (d) usage. **P < 0.01

To study the mechanism underlying circFOXP1 promoting the angiogenesis of osteosarcoma, we further explored the regulatory effect of circFOXP1 on CDKN2AIP, and found that both CDKN2AIP mRNA (Figure 3a) and protein (Figure 3b) expression decreased after inhibiting circFOXP1 expression in osteosarcoma. Moreover, the capacity of tube formation was observed to decline after the usage of CDKN2AIP siRNA (Figure 3c) and circFOXP1 siRNA (Figure 3d) both in U2OS and MG-63 cells. These results showed that circFOXP1 could promote the angiogenesis by increasing CDKN2AIP expression in osteosarcoma.

### circFOXP1 could have competitive combination of miR-127-5p

**Figure 4.**
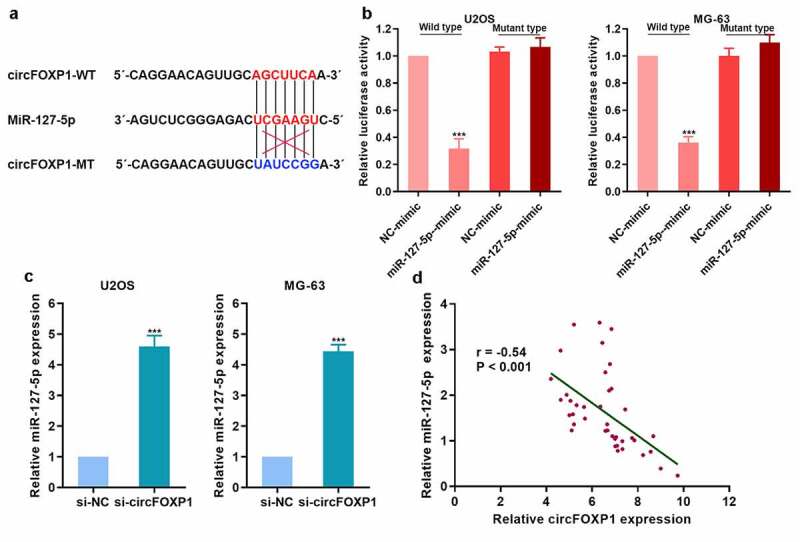
circFOXP1 could have competitive combination of miR-127-5p. (a) The diagram of the binding site of circFOXP1 and miR-127-5p, and the mutation site of circFOXP1. (b) The combination of circFOXP1 and miR-127-5p was confirmed using luciferase reporter assay. (c) miR-127-5p expression in osteosarcoma cells was detected by qRT-PCR after circFOXP1 inhibition. (d) miR-127-5p expression in osteosarcoma was detected, and the correlation between miR-127-5p and circFOXP1 was analyzed. ***p < 0.001

To explore the competitive binding of miRNA to circFOXP1, we found that circFOXP1 could bind miR-127-5p (Figure 4a). We further confirmed that circFOXP1 could bind to miR-127-5p with luciferase reporter assay (Figure 4b). miR-127-5p expression in osteosarcoma cells was upregulated with inhibited circFOXP1 expression (Figure 4c). Then, we detected miR-127-5p expression in clinical tumor tissues, and analyzed its correlation with circFOXP1 expression, and found that miR-127-5p was negatively correlated with circFOXP1 (Figure 4d). Therefore, circFOXP1 might play a role due to competitive adsorption of miR-127-5p.

### miR-127-5p could directly target CDKN2AIP

**Figure 5.**
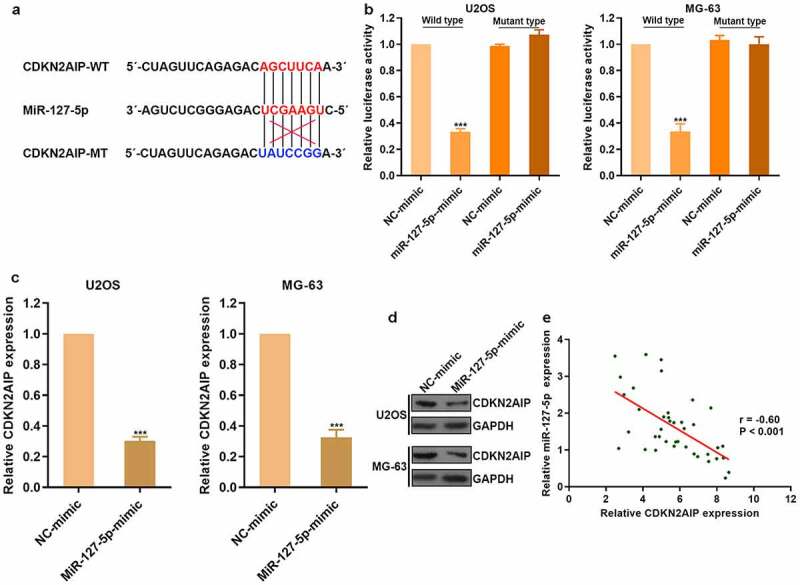
miR-127-5p could directly target CDKN2AIP. (a) The binding site of miR-127-5p with CDKN2AIP and the mutation site of CDKN2AIP were shown. (b) CDKN2AIP was the target of miR-127-5p by luciferase reporter assay. (c) The expression of CDKN2AIP mRNA was detected by qRT-PCR after the treatment of miR-127-5p mimic. (d) CDKN2AIP expression in osteosarcoma was detected, and the correlation between CDKN2AIP and miR-127-5p was analyzed. ***p < 0.001

There was a predicted binding site between miR-127-5p and CDKN2AIP through bioinformatics research (Figure 5a). To clarify the regulatory effect of miR-127-5p on CDKN2AIP, luciferase reporter assay was conducted (Figure 5b). CDKN2AIP expression was obviously reduced after the treatment of miR-127-5p mimic in osteosarcoma (Figure 5c-d). Then, we detected CDKN2AIP expression in clinical tumor tissues and found that miR-127-5p was negatively correlated with CDKN2AIP (Figure 5e). Therefore, circFOXP1 could promote angiogenesis by regulating miR-127-5p/CDKN2AIP signaling pathway in osteosarcoma.

## Discussion

Osteosarcoma, originating in bone, is very common and has quite high metastatic potential. Angiogenesis has been reported to play a vital role in sustained metastasis of osteosarcoma. So, more and more attention has been focused on antiangiogenic therapy, hoping to provide effective therapies for osteosarcoma [16]. For example, Bo Bai et al. declared that a natural compound, with the name of Bavachinin, had potent antiangiogenic ability *in vitro and in vivo*, and could be used as the therapeutic agent to inhibit angiogenesis [17]. Moreover, XH Hu et al. found that bevacizumab exhibited strong antiangiogenic potential *in vivo*, and might be considerable for the treatment in osteosarcoma [18]. In our study, we identified that circFOXP1 could promote angiogenesis by regulating miR-127-5p/CDKN2AIP signaling pathway in osteosarcoma. Our findings provide the novel insight for targeting angiogenesis in osteosarcoma.

Actually, angiogenesis is regulated by the pro-angiogenic and anti-angiogenic factors, as the result of the equilibrium between these factors. The pro-angiogenic factors have been commonly detected to be overexpressed in osteosarcoma. For example, connective tissue growth factor (CCN2) could promote angiogenesis by increasing angiopoietin 2 expression, which served as a key regulator in angiogenesis [19]. The anti-angiogenic factors have been reported to be downregulated in osteosarcoma, such as troponin I and pigment epithelium-derived factor (PEDF) [20]. Recently, miRNAs have been found to play vital roles in angiogenesis. As a typical example, miR-29s could take part in the regulation of angiogenesis through modifying insulin-like growth factor 1 (IGF1) expression [21]. Likewise, there have been a large number of studies showing that circRNAs have a strong ability to regulate angiogenesis recently. The regulation of angiogenesis by circRNAs has been considered to be a quite significant therapeutic strategy for angiogenesis-related diseases. In NSCLC, circABCB10 promoted angiogenesis by acting as a sponge of miR-1252 and interacting with FOXR2 [22]. circVAPA and circRAPGEF5 were found to be obviously overexpressed in tumor and played an important role in promoting angiogenesis [23] [24]. Due to their possible roles in the modulation of angiogenesis, the aspects of circRNA regulation in tumors deserve attention.

However, the mechanisms of circRNAs involved in the regulation of angiogenesis still need to be further elucidated. In our study, we found that circFOXP1 had upregulated expression in osteosarcoma, and higher circFOXP1 expression was closely related to poorer prognosis. Moreover, circFOXP1 showed the powerful ability to promote angiogenesis in osteosarcoma. In mechanism, regulation of CDKN2AIP and circFOXP1/miR-127-5p/CDKN2AIP axis were involved. CDKN2AIP was reported to be pertinent to cell fate and has two-way effects on cancer cells. On the one hand, CDKN2AIP was overexpressed in an ARF-dependent or independent manner to activate p53 function, which was known to be a tumor suppressor [25]. On the other hand, CDKN2AIP overexpression was confirmed to enhance malignant properties in cancer cells [24], and could serve as the marker involved in EMT and invasion in cancer [26]. In our study, we found that CDKN2AIP was obviously upregulated in osteosarcoma and was positively correlated to angiogenesis. Thus, circFOXP1/miR-127-5p/CDKN2AIP regulation axis plays an essential role in osteosarcoma angiogenesis. The use of small RNA molecules has always been a relevant area of investigation with direct clinical application [27–29]. Our results might propose a potential therapeutic target for osteosarcoma. Also, our study provides a new supplement to the pathogenesis of angiogenesis modulated by circRNA in tumor.

## Conclusions

circFOXP1 plays a crucial role in promoting angiogenesis in osteosarcoma. Higher circFOXP1 expression was detected in osteosarcoma and was closely associated with poor prognosis. Moreover, high circFOXP1 expression could promote tumor growth and angiogenesis in osteosarcoma. In terms of mechanisms, circFOXP1 could upregulate CDKN2AIP expression and competitively combinate to miR-127-5p. Furthermore, CDKN2AIP was proved to be directly targeted by miR-127-5p. Therefore, our findings highlighted that circFOXP1 could promote angiogenesis by regulating miR-127-5p/CDKN2AIP signaling pathway in osteosarcoma. Further studies in a large external cohort of osteosarcoma populations are needed to verify our findings.

## Highlight

- Circular RNA CircFOXP1 expression is elevated in osteosarcoma tissues compared to that in adjacent non-cancerous tissues.
- CircFOXP1 promotes the growth and angiogenesis of osteosarcoma through miRNA-127-5p/CDKN2AIP regulation.
- CircFOXP1expression can predict overall survival in osteosarcoma patients.
